# No Difference Between Anchorless and Traditional Suture Anchors in Arthroscopic Bankart Repair: A Clinical Comparison

**DOI:** 10.7759/cureus.26988

**Published:** 2022-07-18

**Authors:** Lucas Haase, Kelsey Wise, Brandon Kelly, John Harris, Jeffrey Macalena

**Affiliations:** 1 Orthopedic Surgery, University Hospitals Cleveland Medical Center, Cleveland, USA; 2 Orthopedic Surgery, University of Minnesota, Minneapolus, USA; 3 Orthopedic Surgery, University of Minnesota, Minneapolis, USA; 4 Orthopedic Surgery, Texas Orthopedic Surgical Associates, Dallas, USA

**Keywords:** comparison, suture anchor, shoulder instability, bankart, shoulder arthroscopy

## Abstract

Background

Shoulder instability and recurrent dislocations are common problems encountered by orthopedic surgeons and are frequently associated with a Bankart lesion. These are classically treated with either open or arthroscopic repair utilizing traditional suture anchors, though anchorless fixation techniques have recently been developed as an alternate fixation method that reduces native bone loss and has comparable pull-out strength.

Methods

A retrospective review was performed at a single institution for patients who underwent Bankart repair from January 2008 through February 2014. American Shoulder and Elbow Surgeons (ASES) questionnaires were mailed to 35 patients with anchorless fixation and 35 age-, gender-, and surgeon-matched patients with traditional suture anchors. Statistical analysis was performed comparing re-dislocation, additional surgery, and ASES scores with statistical significance set at p < 0.05.

Results

Eleven patients in the anchorless implant group and 15 patients in the anchor group completed the questionnaire. The mean follow-up was 4.1 years in the anchorless group and 5.6 years in the anchor group (p=0.04). The number of implants was 4.82 in the anchorless group and 3.87 in the anchor group (p = 0.04). No difference was found in re-dislocation rates (p = 0.80) or additional surgery on the affected shoulder (p = 0.75). ASES scores were found to have no statistical difference (89.89 for the anchorless group versus 85.37 for the anchor group; p = 0.78).

Conclusion

In patients undergoing arthroscopic Bankart lesion repair with traditional anchors compared to anchorless fixation, there appears to be no difference in shoulder re-dislocation rates, recurrent ipsilateral shoulder surgery, or ASES scores.

## Introduction

Shoulder instability and recurrent shoulder dislocations are common problems encountered by orthopaedic surgeons in many different populations, including athletes, so-called ‘weekend warriors’, and trauma patients [1-6]. Anterior shoulder instability, with a Bankart lesion (anteroinferior labrum tear), remains the most common direction and pathology associated with instability. It is typically treated with either non-operative means, specifically physical therapy, or operatively, most commonly with either open or arthroscopic Bankart lesion repair [2,3,5]. Currently, there is discussion over the utility of open versus arthroscopic stabilization, with pros and cons for each [7-9]. With the frequency of these instability events, repair using traditional suture anchors has become a very common procedure but has reported failure rates ranging from 4% to 18% [8,10-12].

In recent years, different implants and techniques have been used in place of these traditional anchors with varying success in Bankart lesions and other labral tears [13,14]. Specifically, anchorless suture fixation has emerged as a promising option for the repair of various soft tissue pathologies [15-18]. Benefits of anchorless suture fixation include smaller drill holes, a reduction in native bone removal, experimentally verified comparable pullout strength to classic suture anchors, and the elimination of the risk of rigid material lost in the joint [19-21]. Although soft anchors have been found to be effective in models, there is a lack of literature on human patients [21]. In 2015, Agrawal et al. reported on the short-term results of triple labrum tears treated with JuggerKnot® devices (Zimmer Biomet, Warsaw, IN) [15]. They found meaningful improvement in patient outcome scores as well as MRI evidence of both labral healing and bone tunnel healing. While these findings are promising, the injuries treated in this study were more complex than most shoulder anterior instability cases. Therefore, the purpose of this study is to assess the clinical outcomes (rate of re-dislocation, reoperation, and American Shoulder and Elbow Surgeons (ASES) Standardized Shoulder Assessment Form score) in a cohort of patients treated arthroscopically with anchorless fixation compared to those treated with traditional suture anchors for Bankart repairs. We hypothesized that the clinical outcomes of the patients undergoing anchorless suture anchor fixation would have equivocal rates of redislocation, reoperation, and ASES scores compared to a group treated with typical suture anchors.

## Materials and methods

After the Institutional Review Board review and approval, a single-institution chart review was conducted on 310 consecutive patients who had previously undergone an arthroscopic Bankart lesion repair between January 2008 and February 2014. There were 35 patients who had repairs that utilized anchorless fixation (JuggerKnot®, Zimmer Biomet, Warsaw, IN). These 35 patients were then age-, gender-, and surgeon-matched to 35 patients who received Bankart lesion repair using traditional suture anchors. Patients were contacted via postal mail to participate in the study. Participation included the completion of an ASES questionnaire as well as questions regarding re-dislocations and further shoulder surgery. Twenty-six total completed questionnaires were returned and scored for this study (11 in the anchorless group and 15 in the matched cohort). Demographic data were obtained as well as the number of anchors or anchorless implants included in the surgical repair. Statistical analysis of the compiled data included Student t-tests comparing re-dislocation, additional surgery, and ASES scores between the two groups (Microsoft® Excel; Microsoft Corp., Redmond, WA). The significance level was set at p < 0.05. There was no external funding used for this study.

## Results

There were 11 patients (10 male, 1 female; mean age of 28.5) in the anchorless implant group, with a mean time from repair of 4.1 years. The control group, with traditional anchors, consisted of 15 patients (13 male, 2 female; mean age of 28), with a mean time from repair of 5.6 years. The follow-up of the traditional anchor group versus the anchorless fixation group was 5.58 years and 4.13 years, respectively. Full demographic data are in Table 1. The anchorless group utilized an average of 4.82 implants per surgery, and the anchor group used an average of 3.87 implants per surgery.

**Table 1:** Demographic characteristics of patients as compared between groups.

	Traditional anchor	Study anchor	P-value
Total patients	15	11	
Male	13	10	0.75
Female	2	1	
Average age	28.03	28.49	0.92
Average follow-up (years)	5.58	4.13	0.04

There were similar numbers of re-dislocations between the two groups (Table 2). In the anchorless group, one patient underwent inferior capsulorraphy for recurrent instability. In the control group, one patient underwent a Latarjet procedure, then subsequently underwent a microfracture and debridement procedure. Another patient in the control group was treated with a hemiarthroplasty.

**Table 2:** Rates of dislocations and subsequent surgeries between groups.

Outcome measure	Traditional anchor	Study anchor	P-value
Re-dislocations (yes/no)	3/15 (20%)	2/11 (18.18%)	0.80
Additional surgeries (yes/no)	2/15 (13.3%)	1/11 (9.1%)	0.75

**Figure 1:**
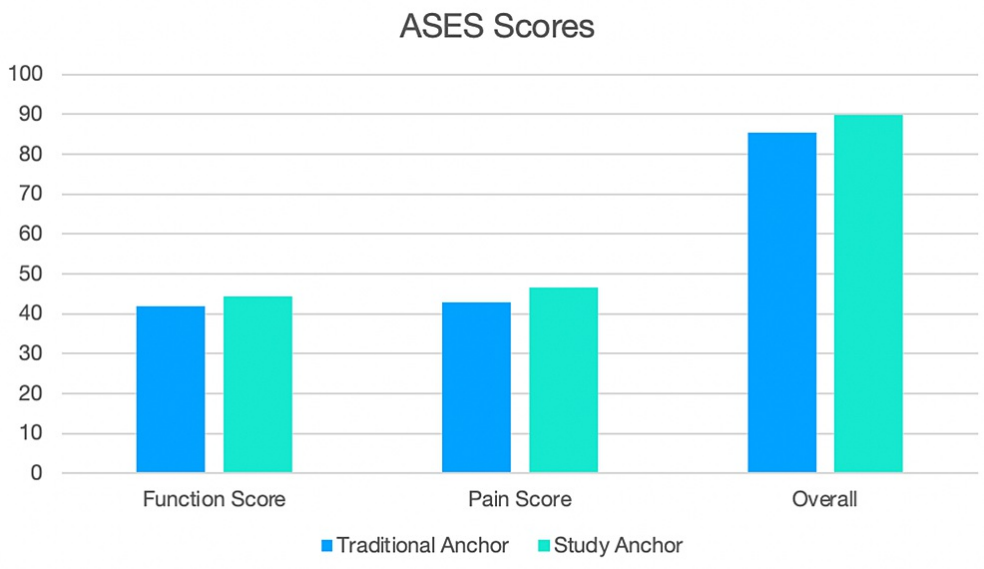
Bar chart depicting the difference in ASES scores between the two groups. ASES: American Shoulder and Elbow Surgeons.

There were similar findings for overall ASES scores between the two groups. Furthermore, there were comparable pain and function subgroup scores (Table 3 and Figure 1).

**Table 3:** Comparison of ASES scores between two groups. ASES: American Shoulder and Elbow Surgeons.

ASES variable	Traditional anchor	Study anchor	P-value
Function score	42.00 (± 7.10)	44.24 (± 6.03)	0.41
Pain score	42.89 (± 9.97)	46.65 (± 9.18)	0.57
Overall	85.37 (± 15.13)	89.89 (± 13.31)	0.78

## Discussion

This study found comparable rates of re-dislocation, reoperation, and outcome scores between anchorless and traditional anchor fixation in arthroscopic Bankart repair. These findings are meaningful as anterior shoulder instability remains a challenge for orthopaedic surgeons, despite the various techniques available to address this pathology. The results of this study are similar to those reported by Lee et al. and Pantekidis et al., who also demonstrated comparable outcome scores and post-operative stability between patients undergoing all-suture anchor bankart repair compared to traditional anchors with an average follow-up of less than 2 years and 28 months, respectively [22,23].

In 2015, Plath et al. reported on 100 shoulders, with an average follow-up of 15 years, treated for anterior stability with arthroscopic Bankart repair. In this case series, they found 21% of patients experienced recurrent instability after fixation [24]. In a systematic review from 2013, Harris et al. found recurrence rates after arthroscopic versus open Bankart repairs to be 11% and 8%, respectively. The average follow-up in their review was 11 years [25]. Other studies report varying recurrence rates, thus providing the impetus for continued research into methods to obtain more reliable long-term outcomes [26].

Anchorless or soft suture devices have been developed and used in many areas in recent years with reports of success comparable to those of traditional fixation devices [18]. Mazzocca et al. performed a cadaveric study that found soft suture fixation devices to be biomechanically similar to traditional suture anchors when used in labral repairs [16]. In 2015, Agrawal et al. reported on the use of the JuggerKnot® all-soft-tissue anchor for the management of triple labrum repairs and found good outcome scores and radiographic healing at approximately two years of follow-up [15]. Despite studies with promising results, concerns about suture devices for Bankart repair also exist. In 2014, Pfeiffer et al. published on the use of two different all-suture glenoid anchors in canine models. Their findings revealed concerns about cyst formation and subsequent failure risks [27]. In a more recent study by Jin and Chun, however, there was no increased rate of peri-anchor cyst formation in those with all-suture anchors and traditional anchors [28]. On the other hand, Iban et al. did find a high rate of peri-anchor cystic changes in a retrospective study of 55 all-suture anchors at one year of use for remplissage. However, this study did not provide a control group for comparison [29]. Further investigation with standardized groups and more common instability patterns, particularly anterior instability, is needed.

There are several limitations to this study. The study is underpowered and the evaluation of a true statistical difference between the groups was not able to be evaluated. The response rate was lower than anticipated, despite diligent follow-up. However, the small sample size was to be expected, given the novelty of using these implants for this procedure. Other limitations include the relatively short follow-up period of approximately four years and the absence of reported clinical examination findings at the final follow-up. Despite these limitations, the data gathered from these small numbers does establish a foundation for the recruitment of subsequent studies with much higher numbers.

The findings in this study should be compared with future studies that incorporate larger numbers of patients with longer follow-ups. If outcomes continue to be comparable, further research should compare the cost between the two techniques, as there is a shifting focus in healthcare on the value of care, which entails maximizing patient outcomes while minimizing financial burden.

## Conclusions

In a small cohort of patients who underwent an arthroscopic Bankart repair using either anchorless or traditional suture anchor implants, there was no apparent difference in recurrent dislocation rate, subsequent ipsilateral shoulder surgery, or ASES scores. The added benefits of smaller drill holes with minimal native bone loss as well as comparable pull-out strength provide an upside to these suture implants. This study should serve as a scaffold for future investigations with greater patient numbers and longer-term follow-ups. Nonetheless, anchorless fixation has promise as an alternative technique in the management of patients with anterior shoulder instability.
